# Optimization of Brucella abortus Protocols for Downstream Molecular Applications

**DOI:** 10.1128/JCM.01894-17

**Published:** 2018-03-26

**Authors:** Noah Hull, Jonathan Miller, David Berry, William Laegreid, Ashley Smith, Callie Klinghagen, Brant Schumaker

**Affiliations:** a Department of Veterinary Sciences, University of Wyoming, Laramie, Wyoming, USA; b Wyoming State Veterinary Laboratory, University of Wyoming, Laramie, Wyoming, USA; University of Tennessee at Knoxville

**Keywords:** brucellosis, DNA concentration, DNA extraction, epidemiology, PCR, molecular methods

## Abstract

We compared the performances of various DNA extraction kits for their ability to recover Brucella abortus strain 19 inoculated into Brucella-free bovine tissues. Tissues were homogenized in a FastPrep bead homogenizer and extracted in triplicate by using one of five kits (Qiagen DNeasy, GE Illustra, Omega Bio-tek E.Z.N.A., Quanta Extracta, and IBI Science DNA Tissue kit). Whole blood was also taken from animals prior to chemical euthanasia, aliquoted, and then fractioned into buffy coat, red blood cells, and plasma. DNA was extracted from whole blood, buffy coat, and plasma by using four kits (Qiagen DNeasy, Omega Bio-tek E.Z.N.A., IBI Science DNA Blood kit, and 5PRIME PerfectPure). Previously reported primers targeting strain 19 were used to amplify extracted DNA and identify the optimal extraction kit. Real-time PCR was performed, and kits were compared for statistical differences by using quantification cycles as an outcome measure. Omega Bio-tek E.Z.N.A. was superior (*P* < 0.0068) in its lower quantification cycle values across all tissue kits. The IBI Science DNA Blood kit was superior to Qiagen DNeasy, 5PRIME PerfectPure, and Quanta Extracta (*P* < 0.0001, *P* = 0.0004, and *P* = 0.0013, respectively) but was not different from Omega Bio-tek E.Z.N.A. (*P* = 1.0). In summary, the optimal extraction kit for B. abortus strain 19 for tissues is Omega Bio-tek E.Z.N.A., and that for blood and its fractions is the IBI Science Mini Genomic DNA kit. Eluted DNA was also concentrated by using the Zymo Research DNA Clean & Concentrator-25 kit. Concentrated eluted DNA with the target was superior (*P* = <0.0001) to unconcentrated eluted DNA.

## INTRODUCTION

While primer and/or probe identification for specific genomic targets in PCR assays is important, the processing of samples, particularly whole tissues, from suspect animals for downstream molecular diagnostics is equally important. The peer-reviewed literature contains multiple studies assessing extractions from “difficult” samples, such as feces and soil, but no data comparing various extraction techniques utilizing commercial kits for Gram-negative bacteria in tissue matrices have been reported (1–3). Our model organism is Brucella abortus, a Gram-negative, nonmotile, facultative, intracellular coccobacillus that is the etiological agent of brucellosis (4). Brucellae are organisms that are known to invade host tissue and reside intracellularly in low numbers (5, 6). Recent Brucella reports have focused on DNA extraction from whole blood, serum, and milk (6, 7). Notably, studies detailing whole-blood and serum extractions are based on human populations. Efficient and relatively inhibitor-free extraction of DNA is critical for use in downstream PCR assays.

Most PCR applications for brucellosis focus on testing postculture isolates from suspected tissues and not directly from tissues of infected animals in the field (8, 9). Most applicably, a study was conducted to determine optimal DNA extraction kits for human serum samples that were spiked with Brucella melitensis vaccine strain Rev 1 (6). However, those spiked human serum samples contained a different, albeit related, organism. Another report details procedures for DNA extraction from laboratory mice challenged with Francisella tularensis (10). That experiment was done with a challenge dose of 100 CFU, where challenged animals were allowed to develop clinical signs prior to euthanasia, sampling, and subsequent DNA extraction. Unfortunately, data from laboratory challenge studies are not analogous to those for clinical specimens typically received in diagnostic laboratories. There is no overlap in kits evaluated in the previous studies and kits evaluated in this study.

According to the World Health Organization, brucellosis is the most widespread zoonosis and is classified as one of the seven most neglected diseases worldwide (11, 12). In the United States, bovine brucellosis, predominantly caused by B. abortus, is the disease of concern due to implications for public health and national and international trade. In the United States, cattle can be infected with B. abortus and Brucella suis. There are no reservoirs of Brucella melitensis (13, 14). Given its proclivity as an intracellular pathogen, B. abortus is well documented for its evasion of the host immune system (15). Bacterial cells are voluntarily sequestered in regional lymph nodes and modulate host immune factors to decrease immune responses (16). Moreover, in infections not temporally concentrated around parturition, B. abortus bacteria persist in low numbers in lymph nodes and lymphoid tissues (17). Thus, bacteriological isolation of this Gram-negative, intracellular pathogen for diagnostics is hampered by this low bacterial burden.

Diagnostic testing for animal brucellosis relies on the “gold standard” of bacteriological culture. Culture is typically carried out on animals that have tested positive upon antemortem assays (i.e., serology). Presumptively positive animals are culled from their respective groups and subjected to necropsy or sampled at slaughter facilities. However, while culture is very specific, it has a low sensitivity. B. abortus can be cultured from only 30 to 50% of seropositive animals, leaving the true status of 50 to 70% of seropositive animals unknown (13). It is unclear whether (i) these 50 to 70% of animals are animals that have cleared the infection and have a long-lasting antibody titer, (ii) these 50 to 70% of animals have been infected with one of several serological cross-reacting organisms, or (iii) culture is unable to detect these low-copy-number organisms in tissues. Therefore, there is a need for diagnostics to move toward more sensitive methods such as PCR. However, preparation of samples for downstream molecular applications is heavily dependent on sample processing and the ability to obtain a target-rich, relatively inhibitor-free template.

Therefore, our objectives were to (i) identify the optimal commercial DNA extraction method for B. abortus and (ii) show DNA concentration methods that increase the quantity of available target DNA for downstream PCR amplification.

## MATERIALS AND METHODS

### Bacterial strains.

Brucella abortus vaccine strain 19 (S19) was used in this study (18). This strain was obtained from the Wyoming Game and Fish Department Wildlife Disease Laboratory (Laramie, WY, USA). Strain 19 was grown on Colombia blood agar plates (Hardy Diagnostics, Santa Maria, CA, USA) at 37°C with 10% CO_2_ for 5 days. Colonies were aseptically collected from the plate and diluted in 4.8 ml of nuclease-free water.

### Blood preparation.

Five 10-ml Vacutainers containing EDTA (Becton Dickinson, Franklin Lakes, NJ, USA) were filled with 7 ml of venous blood (after sedation and before chemical euthanasia, via jugular venipuncture). Blood was spiked with 1 ml of an S19 suspension at 1.87 × 10^7^ CFU/ml and incubated for 24 h at 39°C. Afer incubation, 400 μl of whole blood was aliquoted and used for DNA extraction. The remaining blood in the Vacutainers was then centrifuged at 3,000 rpm at 20°C for 15 min in a Beckman Coulter Allegra 6R centrifuge (Beckman Coulter, Brea, CA, USA). Plasma, buffy coat, and red blood cells (RBCs) were aliquoted following centrifugation, and 400-μl aliquots each were subsequently taken in triplicate for extraction.

### Tissue preparation.

Spleen, cervix, uterus, placentome, and supramammary, prescapular, internal iliac, and medial retropharyngeal lymph nodes from cattle (Bos taurus) were aseptically acquired at the Wyoming State Veterinary Laboratory from diagnostic cases originating outside the region where brucellosis is endemic. Lymph nodes were left intact, while other tissues were taken in 100-g samples. Tissues were inoculated with S19 (1.87 × 10^7^ CFU/ml) by using a 22-gauge needle with a 3-ml syringe. Ten 100-μl injections of the inoculum were made in different locations of the intact tissue, for a total inoculation volume of 1 ml. Tissues were incubated in a humidified incubator at 39°C for 24 h prior to homogenization. Tissues were incubated for 24 h to allow the bacterial cells to infect the tissues in an intracellular manner, analogous to natural Brucella infection. In a biosafety cabinet, three ∼1-g tissue pieces were aseptically collected and placed into 2.0-ml FastPrep tubes (MP Biomedicals, Santa Ana, CA, USA). Included in the tube were 0.10 g of 0.1-mm zirconia-silica beads, 0.28 g of 0.5-mm zirconia-silica beads, and 0.30 g of 1.0-mm zirconia-silica beads (Biospec Products, Bartlesville, OK, USA) in addition to 250 μl of 1× phosphate-buffered saline (pH 7.4). Tubes were placed into the Thermo Savant FastPrep FP120 instrument and run for two 30-s intervals at a speed setting of 4.5. Tubes were then centrifuged in an Eppendorf 5415D microcentrifuge (Eppendorf, Hamburg, Germany) at 12,000 × *g* for 3 min. After centrifugation, 400 μl of the supernatant from the homogenate was placed into a sterile 1.5-ml microcentrifuge tube and extracted with one of the commercial DNA extraction kits. Tissues were processed in triplicate for each kit. All animal work was approved by the University of Wyoming Institutional Animal Care and Use Committee (protocol no. 20140424BS00094-02), and all laboratory work was approved by the University of Wyoming Institutional Biosafety Committee (registration no. 20140630-60).

### DNA extraction kits.

Kits were differentiated based on suggested matrices (tissue or blood). Tissue kits included the Qiagen DNeasy (catalog no. 69506; Qiagen, Hilden, Germany), GE Illustra (catalog no. 28904275; GE, Boston, MA, USA), Omega Bio-tek E.Z.N.A. Tissue (catalog no. D3396-02; Omega Bio-tek, Norcross, GA, USA), Quanta Extracta DNA Prep for PCR (catalog no. 95091-250; Quanta Biosciences, Beverly, MA, USA), and IBI Science Mini Genomic DNA Tissue (catalog no. IB47222; IBI Science, Peosta, IA, USA) kits. Blood kits included the Qiagen DNeasy kit (catalog no. 69506; Qiagen, Hilden, Germany) for whole blood, plasma, buffy coat, and RBCs; the 5PRIME GmbH PerfectPure DNA kit (catalog no. 2302100; 5PRIME GmbH, Hilden, Germany) for whole blood, plasma, buffy coat, and RBCs; Quanta Extracta DNA Prep for PCR (catalog no. 95091-250; Quanta Biosciences, Beverly, MA, USA) for plasma and buffy coat; the IBI Science Mini Genomic DNA Blood kit (catalog no. IB47202; IBI Science, Peosta, IA, USA) for whole blood, plasma, buffy coat, and RBCs; and the Omega Bio-tek E.Z.N.A. Blood DNA minikit (catalog no. D3392-02; Omega Bio-tek, Norcross, GA, USA) for whole blood, plasma, buffy coat, and RBCs. All blood and tissue extraction kits, except for Quanta Extracta, were based on silica spin column technology; Quanta Extracta DNA Prep for PCR is an enzyme digestion extraction kit. Quanta Extracta was indicated for use with buffy coat and plasma samples only. Blood samples, in addition to tissue samples, were run in triplicate. All sets of extractions were run with an extraction control consisting of kit reagents with no biological sample. This served to indicate either kit or environmental contamination. The manufacturers' protocols were followed for each kit, and eluted DNA was stored at −20°C for further analysis.

### Quantification of DNA concentration and purity.

Each triplicate of eluted DNA from the respective kits was assessed for concentration (*A*_260_; bichromatic absorbance correction of 320 nm) and purity (by measure of the *A*_260_/*A*_280_ ratio) on a NanoDrop 2000C instrument (Thermo Fisher Science, Waltham, MA, USA). The NanoDrop 2000C instrument was rezeroed against the elution buffer after triplicates for each kit were analyzed. Interpretation of DNA purity was based on an optimal *A*_260_/*A*_280_ ratio of 1.8 (19).

### Real-time PCR amplification.

Since the samples were spiked bovine tissues and blood, total S19 DNA was not quantifiable by the NanoDrop instrument, as host genomic DNA was also purified. Therefore, S19-specific quantification was required to elucidate optimal extraction kits. Extracted DNA from S19 was amplified by using previously reported primers targeting the erythritol catabolism (*eryC*) gene (20, 21). The *eryC* gene contains a 702-bp deletion in S19 and produces a 361-bp amplicon specific for S19. Primers were ordered from Integrated DNA Technologies (Coralville, IA, USA) and were forward primer 5′-TTGGCGGCAAGTCCGTCGGT-3′ and reverse primer 5′-CCCAGAAGCGAGACGAAACG-3′. A Bio-Rad CFX 96 Touch quantitative PCR (qPCR) thermocycler (Bio-Rad, Hercules, CA, USA) was used to amplify target DNA under the following conditions: an initial denaturation step at 98°C for 5 min, a denaturation step at 95°C for 15 s, an annealing step at 60°C for 15 s, and an extension step at 60°C for 45 s for 40 cycles. The reaction mixture was composed of 1 μl of 20 μM each forward and reverse primer (final concentration of 1 μM each), 2× (10 μl) Bio-Rad iTaq Universal SYBR green mix (Bio-Rad, Hercules, CA, USA), 1 μl of the DNA template, and 7 μl of nuclease-free water for a total reaction mixture of 20 μl. Extraction controls (absence of the biological homogenate) were run with the extraction kits to ensure that the kit components were not contaminated. No-template controls using nuclease-free water as the template were used in the PCR to ensure the absence of environmental or PCR reagent contaminants. DNA extracted from S19 colony isolates was used as a positive control. qPCR thresholds were automatically determined by using Bio-Rad CFX Manager software (version 3.1), utilizing a single threshold mode.

Melting curve analysis was performed after amplification. The hold time prior to melting curve analysis was 95°C for 5 s, followed by 65°C for 5 s, with an increase to 95°C in 0.5°C increments. SYBR green fluorescence curves were analyzed with Bio-Rad CFX Manager software (version 3.1). The melt peak was confirmed based on the melt peak of the positive control, S19.

### DNA concentration/enrichment.

Strain 19 was grown on Colombia blood agar plates (Hardy Diagnostics, Santa Maria, CA, USA) at 37°C with 10% CO_2_ for 5 days. Colonies were aseptically collected from the plate and diluted in 4.8 ml of nuclease-free water. The strain 19 culture suspension aliquot was vortexed, and 400 μl was pipetted into 1.5-ml Eppendorf microcentrifuge tubes (Eppendorf, Hamburg, Germany). This suspension was extracted with the Omega Bio-tek E.Z.N.A. kit with a final elution volume of 400 μl. Eluted DNA from extraction kits was purified and concentrated by using the Zymo Research DNA Clean & Concentrator-25 kit (Zymo Research, Irvine, CA, USA). Two hundred fifty microliters of the eluted DNA was concentrated to 25 μl. All sets of eluted DNA were run with a concentration control, to indicate either kit or environmental contamination. The manufacturers' protocols were followed, and concentrated DNA was stored at −20°C for further analysis.

### Statistical analysis.

Extraction kits were compared by quantification cycle (*C_q_*) values, with lower *C_q_* values indicating more-efficient amplification, which, in this design, we interpreted as an effect of the extraction method. Mean values were calculated based on triplicate runs after confirmation of amplicon size by melting curve analysis. All statistical tests were performed by using JMP Pro version 12.0.1 (SAS Institute, Cary, NC, USA). Statistically significant differences between tissue values were determined by a Kruskal-Wallis test at an alpha value of 0.05. For blood, Kruskal-Wallis statistics included a blocking factor of replicate number. The differences between unconcentrated and concentrated DNAs by the Zymo Research DNA Clean & Concentrator-25 kit were compared by a Wilcoxon signed-rank test. Statistical significance was determined as a *P* value of <0.05.

## RESULTS

### Performance of blood DNA extraction kits.

The DNA concentrations (*A*_260_), DNA purities (*A*_260_/*A*_280_ ratios), and 95% confidence intervals for blood kits are presented in Table 1. For whole blood, 5PRIME PerfectPure had the highest concentration of DNA (54.23 ng/μl), with an acceptable purity value of 1.90. In the plasma fraction of spiked samples, Quanta Extracta had a high DNA concentration (136.33 ng/μl) but poor purity (*A*_260_/*A*_280_ ratio of 0.66), indicating protein contamination. This trend was also seen for the buffy coat sample, with Quanta Extracta having the highest DNA concentration (295 ng/μl) but poor purity (*A*_260_/*A*_280_ ratio of 0.97). The second best commercial kit for buffy coat, when prioritizing DNA purity, was the IBI Science Mini Genomic DNA Blood kit, which had a purity value of 1.79 and a DNA concentration of 35.1 ng/μl. For red blood cells, Qiagen DNeasy appeared to be optimal, with the second highest DNA concentration (12.06 ng/μl) and a purity value of 1.946.

**TABLE 1:** DNA concentrations, DNA purities, and 95% confidence intervals for blood, plasma, buffy coat, red blood cells, and tissues^*a*^

Sample type	Kit	DNA concn (ng/μl) (95% CI)	DNA purity (*A*_260_/*A*_280_ ratio) (95% CI)
Whole blood	Qiagen DNeasy	8.5 (8.2–8.8)	1.92 (1.78–2.06)
	5PRIME PerfectPure	54.23 (38.2–70.3)	1.9 (1.89–1.90)
	IBI Mini Genomic DNA Blood	8.27 (7.3–9.2)	1.79 (1.50–2.08)
	Omega Bio-tek E.Z.N.A.	15.07 (9.1–21.1)	1.79 (1.59–1.99)
Plasma	Qiagen DNeasy	4.73 (4.3–5.2)	1.96 (1.68–2.24)
	5PRIME PerfectPure	2.466 (2.2–2.7)	1.6133 (0.71–2.51)
	Quanta Extracta	136.33 (17.4–255.3)	0.66 (0.63–0.69)
	IBI Mini Genomic DNA Blood	3.533 (1.8–5.3)	1.6733 (0.11–2.26)
	Omega Bio-tek E.Z.N.A.	6.36 (6.2–6.6)	1.6133 (1.18–2.05)
Buffy coat	Qiagen DNeasy	50.63 (26.7–74.5)	1.74 (1.66–1.82)
	5PRIME PerfectPure	99.53 (65.2–133.9)	1.35 (0.97–1.73)
	Quanta Extracta	295 (270.3–319.7)	0.97 (0.96–0.98)
	IBI Mini Genomic DNA Blood	35.1 (32.6–37.6)	1.79 (1.38–2.20)
	Omega Bio-tek E.Z.N.A.	33.6 (24.0–43.2)	1.73 (1.65–1.81)
Red blood cells	Qiagen DNeasy	12.06 (8.7–15.4)	1.946 (1.76–2.13)
	5PRIME PerfectPure	6.366 (6.1–6.7)	2.1266 (2.05–2.20)
	IBI Mini Genomic DNA Blood	11.8 (10.6–13.0)	1.6733 (1.34–2.00)
	Omega Bio-tek E.Z.N.A.	15.76 (14.6–16.9)	1.936 (1.89–1.98)
Tissues (collapsed)	Qiagen DNeasy	103.38 (83.7–123.1)	1.983 (1.96–2.05)
	Quanta Extracta	1797.5 (1797.4–1797.5)	1.566 (1.55–1.59)
	IBI Mini Genomic DNA Tissue	279.37 (263.7–295.1)	1.894 (1.88–1.92)
	Omega Bio-tek E.Z.N.A.	308.77 (253.3–364.2)	1.882 (1.87–1.91)
	GE Illustra	315.22 (308.9–321.6)	1.91 (1.79–2.17)

a CI, confidence interval.

Real-time PCR was performed by using S19-specific primers to elucidate which kit yielded the lowest *C_q_* values in a SYBR green real-time PCR format. These data are included in Table 2. For buffy coat, the sample that contains the highest concentrations of target phagocytes of Brucella, the best-performing kit based on *C_q_* values was the IBI Science Mini Genomic DNA Blood kit (*C_q_* value of 27.72), closely followed by 5PRIME PerfectPure (*C_q_* value of 27.73). For whole blood, the Omega Bio-tek E.Z.N.A. kit had the lowest average *C_q_* (26.16), followed by the IBI Science Mini Genomic DNA Blood kit (*C_q_* of 27.41). For plasma, Omega Bio-tek E.Z.N.A. was superior (*C_q_* of 26.6), followed by the IBI Science Mini Genomic DNA Blood kit (*C_q_* of 27.04). For red blood cells, the IBI Science Mini Genomic DNA Blood kit was optimal (*C_q_* of 27.24). All extraction controls were negative by PCR (*C_q_* of >40).

**TABLE 2:** PCR assay with *C_q_* values of kits based on various matrices and 95% confidence intervals

Sample type	Kit	*C_q_* value	95% CI
Whole blood	Qiagen DNeasy	30.36	29.74–30.99
	5PRIME PerfectPure	28.45	28.15–28.76
	IBI Mini Genomic DNA Blood	27.41	26.93–27.89
	Omega Bio-tek E.Z.N.A.	26.16	24.46–27.86
Plasma	Qiagen DNeasy	28.72	27.87–29.57
	5PRIME PerfectPure	29.7	29.40–30.00
	Quanta Extracta	28.02	25.79–30.24
	IBI Mini Genomic DNA Blood	27.04	26.74–27.34
	Omega Bio-tek E.Z.N.A.	26.6	26.06–27.14
Buffy coat	Qiagen DNeasy	28.39	27.56–29.21
	5PRIME PerfectPure	27.73	26.65–28.82
	Quanta Extracta	29.98	29.33–30.64
	IBI Mini Genomic DNA Blood	27.72	27.26–28.18
	Omega Bio-tek E.Z.N.A.	28.19	26.92–29.45
Red blood cells	Qiagen DNeasy	30.81	30.28–31.34
	5PRIME PerfectPure	28.48	27.85–29.10
	IBI Mini Genomic DNA Blood	27.24	26.91–27.57
	Omega Bio-tek E.Z.N.A.	31.13	30.31–31.95
Tissues (collapsed)	Qiagen DNeasy	26.67	25.88–27.46
	Quanta Extracta	30.07	28.61–31.54
	IBI Mini Genomic DNA Tissue	25.62	24.96–26.29
	Omega Bio-tek E.Z.N.A.	23.94	23.00–24.88
	GE Illustra	29.59	28.73–30.44

There were no statistical differences in the fractions of blood. Therefore, blood and its fractions were concatenated, and a Kruskal-Wallis test was run with the blocking factor of individual replicate. For the concatenated blood, the IBI Science Mini Genomic DNA Blood kit was superior to all other kits (*P* = 0.0013), except for the Omega Bio-tek E.Z.N.A. kit, which showed no difference (*P* = 1.0).

### Performance of tissue DNA extraction kits.

The DNA concentrations (*A*_260_), DNA purities (*A*_260_/*A*_280_ ratios), and 95% confidence intervals for tissue kits are presented in Table 1. Quanta Extracta appeared to be inferior when evaluating DNA purity. However, the Omega Bio-tek E.Z.N.A., IBI Science Mini Genomic DNA Tissue, Qiagen DNeasy, and GE Illustra kits were similar within individual tissues (*P* = 0.83). Therefore, to increase power, all tissues were collapsed and evaluated as a whole. Quanta Extracta had the highest average DNA concentration (1,797.5 ng/μl) but the lowest purity (*A*_260_/*A*_280_ ratio of 1.566). GE Illustra had the second highest DNA concentration (315.22 ng/μl), with a purity of 1.91. This was followed by similar quantification with the Omega Bio-tek E.Z.N.A. kit, with a DNA concentration of 308.77 ng/μl and a purity of 1.882.

Real-time PCR was performed by using S19-specific primers to elucidate which kit yielded the lowest *C_q_* value in a SYBR green real-time PCR format. Tissue types were concatenated by kit. The optimal kit by *C_q_* value was the Omega Bio-tek E.Z.N.A. kit (*C_q_* of 23.94), followed by the IBI Science Mini Genomic DNA Tissue kit (*C_q_* of 25.62), Qiagen DNeasy (*C_q_* of 26.67), GE Illustra (*C_q_* of 29.59), and Quanta Extracta (*C_q_* of 30.07). All extraction controls were negative by PCR (*C_q_* of >40).

For concatenated tissues, a Kruskal-Wallis test without a blocking factor was run to determine the likelihood of a type I error. Statistical associations are represented in Tables 3 and 4. For concatenated tissues, the Omega Bio-tek E.Z.N.A. kit was superior to all kits against which it was tested (*P* = 0.0068).

**TABLE 3:** Kit-versus-kit statistical analysis to determine the optimal extraction kit for blood^*f*^

Kit for blood (collapsed)	*P* value				
	IBI	Qiagen	5PRIME	Omega	Quanta
IBI^*a*^		**<0.0001**	**0.0004**	1	**0.0013**
Qiagen^*b*^			0.0689	0.0782	0.7506
5PRIME^*c*^				0.2145	**0.0193**
Omega^*d*^					0.0997
Quanta^*e*^					

a Directionality based on a *C_q_* value of 27.35.

b Directionality based on a *C_q_* value of 29.57.

c Directionality based on a *C_q_* value of 28.59.

d Directionality based on a *C_q_* value of 28.02.

e Directionality based on a *C_q_* value of 29.32.

f A Kruskal-Wallis test was conducted on concatenated blood. For blood, a blocking factor on replicate number was employed. *P* values in boldface type indicate significance at a *P* value of <0.05.

**TABLE 4:** Kit-versus-kit statistical analysis to determine the optimal extraction kit for tissue samples^*f*^

Kit for tissue (collapsed)	*P* value				
	Omega	IBI	Qiagen	GE	Quanta
Omega^*a*^		**0.0031**	**0.0002**	**<0.0001**	**0.0068**
IBI^*b*^			0.0568	**<0.0001**	**0.0064**
Qiagen^*c*^				**<0.0001**	**0.0228**
GE^*d*^					0.4636
Quanta^*e*^					

a Directionality based on a *C_q_* value of 23.94.

b Directionality based on a *C_q_* value of 25.62.

c Directionality based on a *C_q_* value of 26.67.

d Directionality based on a *C_q_* value of 29.59.

e Directionality based on a *C_q_* value of 30.07.

f A Kruskal-Wallis test was conducted on concatenated tissue samples. Tissues did not have a blocking factor, as all samples were independent. *P* values in boldface type indicate significance at a *P* value of <0.05.

### Performance of DNA concentration/enrichment.

Preconcentrated DNA had a mean concentration of 1.32 ng/μl with a purity of 1.66. After concentration using the Zymo Research DNA Clean & Concentrator-25 kit, the mean concentration was 13.37 ng/μl, with a purity of 1.92. Before concentration, the average *C_q_* value was 37.33, which was reduced to 32.54 after concentration using the Zymo Research kit. Results are shown in Table 5. Postconcentration *C_q_* values were lower (*P* < 0.0001) than preconcentration *C_q_* values, indicating that there was more target DNA in the template. This was consistent with the DNA concentration being higher (*P* < 0.0001) in postconcentration samples than in preconcentration samples. All concentration controls for the Zymo Research kit were negative by PCR (*C_q_* of >40).

**TABLE 5:** DNA concentration and DNA purity before concentration versus after concentration with the Zymo Research DNA Clean & Concentrator-25 kit^*a*^

Parameter	Value (95% CI)		*P* value
	Preconcentration	Postconcentration	
DNA concn (ng/μl)	1.32 (1.22–1.43)	13.37 (9.08–17.66)	<0.0001
DNA purity (*A*_260_/*A*_280_ ratio)	1.66 (1.49–1.82)	1.92 (1.81–2.03)	<0.0001
*C_q_*	37.33 (36.67–37.98)	32.54 (30.16–34.91)	0.0155

a A Kruskal-Wallis test was performed on DNA concentration (*A*_260_), DNA purity (*A*_260_/*A*_280_ ratio), and real-time PCR *C_q_* values with 95% confidence intervals preconcentration versus postconcentration. All *P* values are significant at a *P* value of <0.05.

## DISCUSSION

Due to the possibility of a low bacterial burden of brucellae, efficient capture of target genomes in various clinical samples is needed to achieve the highest possible sensitivity in diagnostics while avoiding false-negative results that can confound diagnostics. The main objectives of this study were to identify optimal commercial DNA extraction kits for use with B. abortus and to identify a DNA concentration method that could capture low-copy-number infections by molecular diagnostics. We quantitatively assessed DNA extraction kits based on DNA concentration (*A*_260_), DNA purity (*A*_260_/*A*_280_), and *C_q_* values on a real-time PCR SYBR green platform. However, since commercial extraction kits indiscriminately purify both pathogen and host genomic DNAs, measurement of the crude DNA concentration is not an ideal metric for selection of the optimal extraction kit. Therefore, direct quantification of S19 target DNA utilizing *C_q_* values determined by PCR was used as the determining factor for kit selection.

The Omega Bio-tek E.Z.N.A. kit is the optimal kit, of those tested, for the extraction of DNA from spiked bovine tissue samples. The Omega Bio-tek E.Z.N.A. kit was the optimal kit for the extraction of DNA from whole blood. For buffy coat, the IBI Science Mini Genomic DNA Blood kit proved to be optimal for the extraction of high quantities of relatively pure DNA. Emphasis was placed on whole blood and buffy coat, as brucellae are known for intracellular infection of phagocytes and thus would be more readily found in these samples than in plasma or red blood cells (22). Additionally, in previous studies, whole blood and buffy coat were found to be optimal clinical samples for culture and PCR for human patients with brucellosis (23). While this is not directly related to chronically infected cattle, no studies have been undertaken to assess the sensitivity of PCR using whole-blood or buffy coat samples to identify infected animals. Additionally, differences in *C_q_* values are directly applicable to veterinary diagnostic laboratory assays. Typically, a delta value of 3 for *C_q_* values roughly corresponds to a log difference of amplicon target numbers (24, 25). Therefore, for kits that have lower *C_q_* values, this would increase the sensitivity of a given PCR assay. In situations where expected target DNA could be present in low copy numbers, concentration of eluted DNA is achievable with the Zymo Research DNA Clean & Concentrator-25 kit. This kit achieved a 10× concentration of the eluted DNA; thus, 1 μl of the template postconcentration is equivalent to ∼10 μl of the original elution volume.

Interestingly, Quanta Extracta consistently had the highest *A*_260_ yet suffered from low DNA purity. This was consistent with real-time PCR results, where samples extracted by Quanta Extracta consistently had the highest *C_q_* values. This kit does not make use of a silica spin column. Therefore, there is no true purification of the sample by this methodology, explaining the protein contamination seen in purity measurements.

Previous studies found that phase separation techniques that rely on protein precipitation followed by DNA precipitation are not optimal (6, 26, 27). Additionally, it is well documented that traces of phenol can completely inactivate *Taq* polymerase, thus complicating downstream applications (28). Phase separation can also be highly dependent on the technical skills of the individuals performing the extraction. The kits evaluated in this study utilized digestion with proteolytic enzymes (proteinase K) to achieve cell lysis. In comparison to phase separation techniques, commercial kits do not utilize hazardous chemicals and can be highly adoptable to a laboratory setting. All spin column kits in our experiment utilized a silica membrane. This technique utilizes enzyme digestion to release nucleic acids from cells, followed by nonspecific nucleic acid absorption to the silica fibers within the membrane. Washes of the spin column with high-salt-concentration buffers strip away low-molecular-weight compounds and residual proteins. Extraction is completed with a low- or no-salt elution buffer, which reverses the nonspecific absorption of nucleic acids from the silica fibers and into the eluate. It was reported previously that a drawback of spin column extraction kits is the potential for cross-contamination due to the aerosolization of other samples during centrifugation steps (6, 29). In our study, we ran extraction and Zymo Research concentration controls on PCR, which allowed us to evaluate cross-contamination, either by aerosolization (centrifuge and pipette, etc.) or by contamination of kit components. All extraction and concentration controls were negative by PCR (*C_q_* of >40).

While the kits tested in this study have not been extensively compared and reported, they have been widely utilized in a variety of studies. The Omega Bio-tek E.Z.N.A. kit was used previously for sampling a multitude of biological samples (30–32). Additionally, the same is true for the IBI Science Mini Genomic DNA Blood and Tissue kits (33, 34). Much has been reported on DNA extraction techniques. Unfortunately, many of those studies dealt with methodologies that predate commercial DNA extraction kits. More recent studies evaluating commercial DNA extraction kits have focused on fungal DNA or “difficult” samples, such as soil, feces, or paraffin-embedded tissues (1, 3, 35, 36). This is the first study to assess the optimal DNA extraction kits for use on a Gram-negative intracellular bacterium from multiple matrix types.

While the capture of target DNA is vital for downstream molecular applications, protocols for the homogenization of tissues for use in commercial DNA extraction kits are equally important. In this study, we used spiked tissue samples, 3 g of which was placed into a FastPrep homogenization tube for bead beating. However, in challenge studies, the bacterial burden within lymph nodes can be as low as 17 bacterial cells per lymph node (17). Therefore, subsampling of 3 g of tissue from a lymph node introduces the risk of missing these bacterial cells for extraction. Newer technologies such as the Omni Bead Ruptor allow the homogenization of whole lymph nodes in 50-ml conical tubes. However, one would have to consider homogenization media for the use of this platform. Certain media, such as garnet-sharp particles, can have a shearing effect on bacterial cells and potentially DNA and can result in a lower sensitivity of bacteriological culture or downstream molecular diagnostic methods (37).

In summary, these results demonstrate that the Omega Bio-tek E.Z.N.A. kit was optimal for whole blood and tissues, while the IBI Science Mini Genomic DNA Blood kit was optimal for buffy coat samples. These kits showed optimal capture of target-specific DNA across inoculated matrices. These kits are easily adoptable within most laboratories and require standard equipment found in most microbiology laboratories. These kits provide high-quality eluates that can then be concentrated by using other commercial kits such as the Zymo Research DNA Clean & Concentrator-25 kit. The most efficient DNA capture methods use commercial kits, followed by concentration of the eluted DNA, which assists in increasing the sensitivity of molecular diagnostics for these intracellular, low-copy-number infections.
